# Estimating the velocity and direction of African Swine Fever spread in wild boar populations in South Korea using Trend-Surface Analysis

**DOI:** 10.1371/journal.pone.0346098

**Published:** 2026-04-02

**Authors:** Cecilia Aguilar-Vega, Jaime Bosch, Satoshi Ito, Benjamin Ivorra, Hyunkyu Jeong, José Manuel Sánchez-Vizcaíno

**Affiliations:** 1 VISAVET Health Surveillance Centre, Complutense University of Madrid, Madrid, Spain; 2 Animal Health Department, Faculty of Veterinary Medicine, Complutense University of Madrid, Madrid, Spain; 3 Current Address: IRTA, Animal Health, Centre de Recerca en Sanitat Animal (CReSA), Campus de la Universitat Autònoma de Barcelona (UAB), Bellaterra, Catalonia, Spain; 4 Current Address: Unitat mixta d’investigació IRTA-UAB en Sanitat Animal, Centre de Recerca en Sanitat Animal (CReSA), Campus de la Universitat Autònoma de Barcelona (UAB), Bellaterra, Catalonia, Spain; 5 South Kyushu Livestock Veterinary Center, Kagoshima University, Soo, Japan; 6 Interdisciplinary Mathematics Institute (IMI) & Department of Applied Mathematics and Mathematical Analysis, Complutense University of Madrid, Madrid, Spain; 7 Dodram Pig Research Center, Daejeon, Republic of Korea; University of Illinois Urbana-Champaign College of Veterinary Medicine, UNITED STATES OF AMERICA

## Abstract

African swine fever (ASF) is a lethal disease of swine that has spread across Asia since its introduction in 2018. South Korea first reported the disease in September 2019 in domestic pigs, and since then, more than 4,000 cases have been reported in wild boars during its expansion up to August 2024. Due to the high number of ASF notifications in wild boars in South Korea, contrasted with their scarcity in most Asian countries, analyzing the spatiotemporal spread of the disease in a setting with active surveillance provides valuable insights. In this study, we performed a trend-surface analysis on temporally gridded case data to characterize the overall geographic spread and direction of ASF in wild boars across South Korea, from its emergence to August 2022. Additionally, we propose a novel approach distinct from previous studies, to estimate spread velocity by incorporating an upper threshold to avoid unrealistic values. The model described the spread of ASF in the study area. The disease showed greater expansion in the east of the country. Initially, a south and eastward direction was estimated. The estimated median velocity was 19.53 km/month, with cell-level velocities ranging from 2.45 to 69.99 km/month. Velocity increased notably from autumn 2021 onward and varied substantially across years. Our results show the dynamics of ASF in wild boars of South Korea, providing new evidence of their role in the epidemiology of the disease.

## Introduction

African swine fever (ASF) is a highly contagious, non-zoonotic infectious hemorrhagic disease that affects both domestic pigs and wild suids. Its etiological agent is the African swine fever virus (ASFV), the only member of the genus *Asfivirus*, family Asfarviridae [1]. Virulent strains can cause 90–100% mortality in naïve animals [2]. The clinical severity of the disease, along with the socio-economic consequences due to control measures and trade restrictions, as well as the lack of effective treatment and vaccine, makes it a notifiable disease to the World Organization for Animal Health, and a major concern for the livestock industry [3].

ASF genotype II spread anthropogenically to Georgia (Europe) in 2007 from Africa [4]. After a remarkable spread in Europe, it was introduced to Asia via China in 2018 [5]. In 2019, the virus spread to ten Asian countries including South Korea. The first notification of the disease in South Korea was in September 2019 in a domestic pig farm located in the northwest, close to the border with North Korea [6,7]. Shortly after, in October, the first ASF case in wild boar was notified less than 40 km from the first domestic pig outbreak [6]. Since then, the disease spread south and east of the country, primarily affecting wild boar [5–7]. Only 26 outbreaks in domestic pigs were reported during the first three years of the epidemic in South Korea, 14 of them occurring in 2019 [7]. In the months following the first confirmation of ASF in the country, farms in close proximity or with an epidemiological connection to infected farms were depopulated [8,9]. Consequently, the majority of ASF cases occurred in wild boar, and domestic pig outbreaks were deemed a spillover [7].

In Europe, wild boar plays a key role in the spread and maintenance of the disease in most affected countries [10]. The role of wild boar in Asia is not well defined due to the lack of surveillance in wild populations of suids. However, in some countries, such as South Korea and Malaysia, as well as Far East Russia on the border with China, ASF cases have been reported in wildlife [5], indicating a high probability of underestimation in the rest of Asian countries [11,12]. In addition, a global study of the distribution of wild boar predicted higher densities in southern Asia compared to Europe [13,14]. Thus, studying the geographical spread of the disease in areas of Asia where wild boar populations are included in ASF surveillance systems, and with few notifications in domestic pigs [7], could be highly beneficial for understanding the expansion of the disease in areas with high-density wild boar populations [7,8]. The foremost goal of this study was to estimate the wave direction and velocity of the spread of ASF in wild boar populations of South Korea, including both natural spread and possible human-mediated transmission.

## Materials and methods

### Study area

For the study, only mainland South Korea was included (approximately 34.3–38.62º N, 125–129.6º E). Tree coverage represents 70% of the country [7], and it is accompanied by a widespread wild boar habitat suitability [15]. The orography of the country is also favorable for wild boar, with a maximum altitude of 1,950 meters above sea level [16,17]. The Taebaek mountain range extends 500 km from the northeast to the southeast of the Korean peninsula along the coast [18]. In some areas of the northern region of the country, wild boar density was estimated based on culling and carcass removal efforts, to be close to 10 animals per km^2^ [14].

The country has a temperate climate with four distinct seasons: spring (March to May), summer (June to August), autumn (September to November), and winter (December to February). Most precipitation occurs in the summer, whereas winters are dry, with only 10% of the country's annual precipitation falling as snow [19].

### Trend-surface analysis and velocity estimation

Trend-surface analysis (TSA) is a method that relies on the application of least-squares regression to geographical locations to generate a surface interpolation. This method uses the “best-fitting” polynomial (linear, quadratic, cubic or higher-order) to fit empirical data using their geographical location (in coordinates (*X*, *Y)*) [20,21]. TSA is a simple but effective technique to identify the direction and speed of an infectious disease [22–24].

The geographical location of ASFV PCR-positive wild boars, notified from the first notification in 2019 and up to August 2022, was provided by the Dodram pig research center, South Korea [25]. No specific permits were required for this study, as the analyses were based exclusively on data in publicly accessible sources, and no additional field sampling or on-site activities were performed.

A grid, with a cell size of 10 km x 10 km, was generated in ArcGIS v10.8.1 for the entire surface of South Korea to avoid the negative effects on the results of cluster point data [26] due to the extended circulation of ASF in an area over time, and to avoid large and uneven administrative spatial units. The origin of the geographical coordinates was adjusted to the cell centroid [22–24], and the date of the first notification was assigned to each grid cell where ASF was notified in wild boar. Certain ASF-affected grid cells were excluded from the analysis because they represented abrupt epidemiological jumps and were identified as spatial outliers that could distort the underlying trend-surface model. We obtained a total of *I* cells. The decimal degree coordinates of the cell centroid (*X*_*i*_, *Y*_*i*_) were transformed into kilometers.

Let T*(x,y)* be the date of the earliest notification of ASF in wild boar at coordinate *(x,y)*. We estimate T*(x,y)* by considering a function of the form T(x,y)=f(x,y)+ u(x,y), where f(x,y) indicates the trend surface and u(x,y) are the residuals for each spatial unit [20].

To select the best fitting model according to the reported data, a backward stepwise selection was applied to the polynomial models. Elements of the model were removed when non-significant (*p* > 0.05), and the model with the best Akaike’s Information Criterion (AIC) and Bayesian Information Criterion (BIC), was chosen. Once the best model was chosen, we verified the absence of violation of regression model assumptions.

For the spatiotemporal representation and posterior velocity analyses, the centroid of a 1 km x 1 km grid, generated exclusively from the affected 10 km x 10 km grid, was used to calculate the predictive values of the final model. Predicted values were used to generate contour maps of the spread of ASF in affected areas of South Korea using the “spline with barriers” tool in ArcGIS v10.8.1. A three-month interval (90 days) was applied for the representation of contour lines to allow sufficient separation for discerning the spread of ASF. To minimize the impact of the “edge effect” phenomenon a buffer zone was generated [20], including ASF-affected districts, municipal cities and counties, and some neighboring southern regions [27]. This phenomenon results from the limited ability of regression models to extrapolate beyond the boundaries of the study area and is particularly pronounced in quadratic and higher-order trend-surface analyses (TSA). Therefore, the model’s predictions in adjacent areas with no data were considered unreliable [20,22], and were excluded from the final output.

The partial derivatives of *T(x,y)* according to coordinates *X* (i.e., ∂T/∂X) and *Y* (i.e., ∂T/∂X) were obtained to calculate the velocity and direction of the spread of ASF in the study area. More precisely, the velocity of the disease in kilometers per month was obtained by considering


$$v(x,y)=\sqrt{{\left(\frac{\partial X}{\partial T}(x,y)\right)}^{\text{2}}+{\left(\frac{\partial Y}{\partial T}(x,y)\right)}^{\text{2}}}$$

where $\frac{\partial X}{\partial T}(x,y)=\frac{\text{1}}{\frac{\partial T}{\partial X}(x,y)}$ and $\frac{\partial Y}{\partial T}(x,y)=\frac{\text{1}}{\frac{\partial T}{\partial Y}(x,y)}$.

The direction of the ASF spread (assuming that 0 degrees is EASTWARD) is given by


$$\theta (x,y)={tan}^{-\text{1}}\left(\frac{\partial Y}{\partial X}(x,y)\right)-\pi \;min\left(\frac{\left|\partial X\right|}{\partial X}(x,y),\text{0}\right),$$

where $\frac{\partial Y}{\partial X}(x,y)=\frac{\frac{\partial T}{\partial X}(x,y)}{\frac{\partial T}{\partial Y}(x,y)}$ and $\frac{\left|\partial X\right|}{\partial X}(x,y)=\frac{\frac{\partial T}{\partial X}(x,y)}{\left|\frac{\partial T}{\partial X}(x,y)\right|}.$

Given the polynomial nature of the model, ∂T/∂X and ∂T/∂X may be close to 0, generating unrealistic high velocities. Therefore, based on expert opinion and scientific literature [28–32], an upper velocity threshold of 70 km/month was established. This value was considered sufficiently high to encompass plausible human-mediated intervention, while velocities exceeding threshold were deemed unrealistic. The aim was to capture both natural disease spread and reasonable human-assisted movements without incorporating extreme anomalies. Additionally, similar velocities have been reported as possible in other ASF spread studies [33]. A sensitivity analysis was performed to assess the impact of the threshold on the overall velocity estimates.

The TSA model and subsequent calculations were performed using R v4.2.2 [34]. We used the following packages for data manipulation, modeling and visualization: “dplyr” [35], “reshape2” [36], “lubridate” [37], “stats” [38], “Metrics” [39], “lmtest” [40], “raster” [41], “sf” [42], “sp” [43], “geosphere” [44], “rnaturalearth” [45], “ggplot2” [46], “lattice” [47], and “gridExtra” [48].

## Results

### Description of ASF notifications

**Fig 1:**
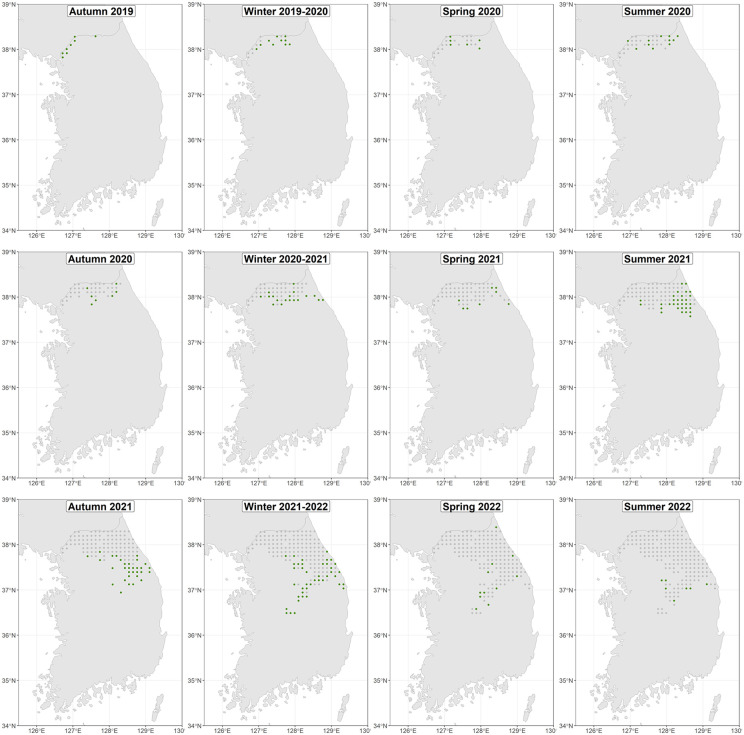
Spread of ASF in wild boar in South Korea from August 2019 until August 2022. Coordinates correspond to the centroid of affected grid cells used for the final model. According to the date of the first case, green dots correspond to the spread of that season, while gray dots represent the previously affected areas.

The database of ASF notifications in wild boar comprised a total of 2,621 cases for the study period (2019 to August 2022). In the 10 km x 10 km grid, 196 ASF-affected grid cells were identified, although 190 were included to generate the final model. The spread of the disease to new areas varied between the years: 11 cells in 2019, 40 in 2020, 87 in 2021, and 52 in the study period of 2022, with the number of affected cells and the distribution of the disease increasing each year. Regarding seasonality, in winter ASF showed greater spatial expansion with 70 new affected grid cells, followed by summer with 49 new cells, autumn with 46 new affected grid cells, and lastly, spring when the disease spread to 25 cells. The spatial distribution of the disease in the gridded study area per season is gathered in Fig 1.

### Trend-surface analysis

The fourth-order TSA model was selected based on the performance of the best order polynomial models using backward stepwise selection (Table 1). The best performing model (Equation 1) improved all metrics regarding the other models, and was of the form (S1 Table):

**Table 1:** Performance of the best model for each order polynomial trend-surface analysis (TSA).

Order	AIC^a^	BIC^b^	R^2^	Adjusted-R^2^	MAE^c^	RMSE^d^
1	1133.52	1146.51	0.73	0.73	3.7	4.68
2	1072.6	1092.08	0.81	0.8	2.98	3.94
3	1038.69	1067.91	0.85	0.84	2.78	3.55
4	996.21	1031.93	0.88	0.87	2.48	3.14

^a^ AIC: Akaike’s Information Criterion.

^b^ BIC: Bayesian Information Criterion.

^c^ MAE: mean absolute error.

^d^ RMSE: root mean square error.


$$T(x,y)=\beta_{\text{0}}+\beta_{\text{1}}y^{\text{2}}-\beta_{\text{2}}xy+\beta_{\text{3}}x^{\text{3}}+\beta_{\text{4}}y^{\text{3}}+\beta_{\text{5}}x^{\text{2}}y-\beta_{\text{6}}x^{\text{4}}+\beta_{\text{7}}y^{\text{4}}-\beta_{\text{8}}x^{\text{3}}y+\beta_{\text{9}}x^{\text{2}}y^{\text{2}}$$ (1)

**Fig 2:**
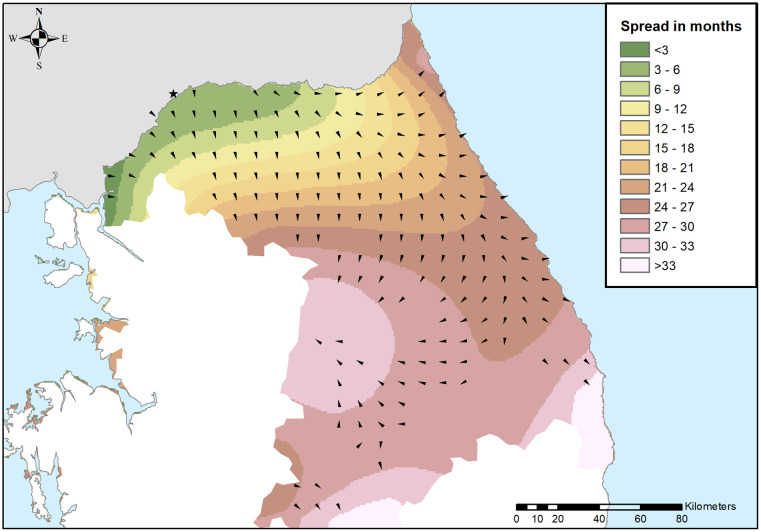
Predicted progress in trimesters of ASF cases in South Korea using a fourth-order polynomial trend-surface analysis. The star marks the grid cell where the first ASF notification in wild boar was reported. Arrows show the direction of each grid cell centroid obtained from the vectors contributing to the slope of disease spread. White areas represent ASF-free administrative units in South Korea until August 2022. Administrative boundaries are republished from [27] under a CC BY 4.0 license.

**Fig 3:**
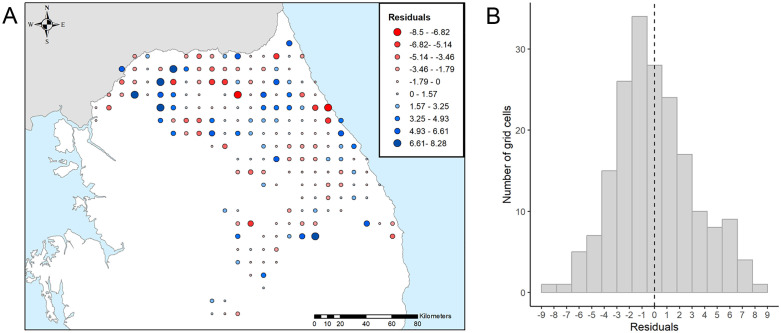
Best fitting model residuals (A), and histogram of best fitting model residual (B). Dots correspond to the centroid of the cell where ASF cases in wild boar were reported. The dashed line in the histogram corresponds with the mean value of residuals. Administrative boundaries are republished from [27] under a CC BY 4.0 license.

Contour lines from the predicted values of the model showed the progress of ASF in the study area (Fig 2). In the early stages of the disease introduction, the spread was towards the south and east of the country. This dispersion pattern continued as the disease progressed. The residuals were normally distributed with a mean of −1.27 x 10^−16^, and a range of −8.50 to 8.28 (Fig 3B). Extreme residual values were scarce, whilst the interquartile range was 3.92 months. To evaluate the areas where the disease occurred before or after the model's prediction, the residuals were plotted (Fig 3A). Positive residuals correspond to areas where ASF occurred after the model's prediction, indicating the disease was delayed. On the other hand, negative residuals show areas where ASF occurred before the prediction of the model, meaning it advanced earlier than predicted.

### Estimation of ASF velocity and direction

To obtain the velocity and direction of the epidemic waves of ASF, the derivatives of *X* and *Y* of the final model (Equation 1) were obtained (Equations 2 and 3):


$$\frac{\partial T}{\partial X}(x,y)=-\beta_{\text{2}}y+{\text{3}\beta }_{\text{3}}x^{\text{2}}+\text{2}\beta_{\text{5}}xy-\text{4}\beta_{\text{6}}x^{\text{3}}-\text{3}\beta_{\text{8}}x^{\text{2}}y+\text{2}\beta_{\text{9}}xy^{\text{2}}$$ (2)

$$\frac{\partial T}{\partial Y}(x,y)=\text{2}\beta_{\text{1}}y-\beta_{\text{2}}x+\text{3}\beta_{\text{4}}y^{\text{2}}+\beta_{\text{5}}x^{\text{2}}+{\text{4}\beta }_{\text{7}}y^{\text{3}}-\beta_{\text{8}}x^{\text{3}}+{\text{2}\beta }_{\text{9}}x^{\text{2}}y$$ (3)

**Fig 4:**
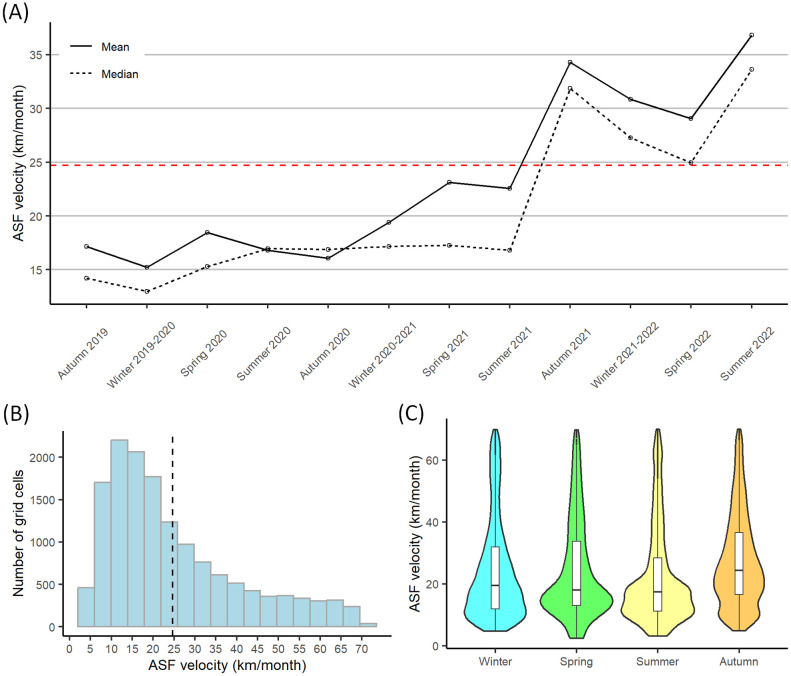
Velocity analysis performed using the 1 km x 1 km grid and the actual date of infection. **(A)** Mean and median velocities each season. The red dashed line corresponds with the mean value of velocities (24.70 km/month). **(B)** Velocity histogram for the ASF-affected grid cells. The black dashed line corresponds with the mean value of velocities. **(C)** Seasonal variations in velocity.

The velocity analysis was performed using the 1 km x 1 km grid, containing 18,229 cells. After the application of the upper velocity threshold of 70 km/month, 14,675 cells remained (80.5%). The velocity of ASF spread varied from 2.45 km/month to values close to the established threshold (69.99 km/month), with a mean of 24.70 km/month and a median of 19.53 km/month. The mean and median estimated velocity for actual dates (expressed as season and year) varied substantially (Fig 4A). In the first and second years of the epidemic, the mean and median speed of the disease was 18.87 and 17.92 km/month in 2019, and 16.28 and 14.47 km/month in 2020, respectively. In 2021 the mean and median increased to 26.65 and 22.78 km/month, and in 2022, they reached 30.99 and 27.47 km/month, respectively. As shown in Fig 4A, until summer 2021, the velocity of spread of the disease remained fairly constant, especially for the median velocity, and mainly below the total mean. From that point on, ASF velocity increased considerably. Some differences between the four seasons were observed (Figs 4A and 4C), they were not substantial enough to draw solid conclusions.

The sensitivity analysis of the upper velocity threshold (Table 2) shows that that the median velocity exhibited less variability than the mean across different threshold values.

**Table 2:** Mean and median ASF velocity with different velocity thresholds.

Velocity (km/month)	300	200	100	90	80	70	60	50	40	30
**Median**	23.49	22.85	21	20.64	20.14	19.53	18.8	18.13	17.18	15.7
**Mean**	41.53	36.73	28.84	27.67	26.32	24.7	22.63	20.6	18.53	16.04

## Discussion

The geographical spread of ASF in wild boar in Asia is difficult to evaluate due to the lack of reporting in most countries [5]. Thus, assessing the spatiotemporal evolution of the disease in wild boar in a country with active surveillance for wild populations could improve the understanding of such spread. Here, the distribution and velocity of ASF in South Korea have been generated and evaluated considering all scenarios of spread. Various studies have already conducted to predict the natural spread of ASF among wild boar populations [49–53]. The spread of the disease in terms of distribution and velocity was different during the study period (Figs 2 and 4A), showing that the dynamics of the disease in wild boar varied at different stages of its expansion.

The pattern of ASF spread presented here can be partially comparable with the spread of ASF in Estonia where the speed of the disease was initially low (up to 3 km/month) and increased to 12 km/month a year into the epidemic [51]. The obtained mean and median velocities could be comparable to the ASF estimated spread in the European Union between 0.6 to 54 km/month, with a mean of 11.28 km/month using a spatio-temporal kriging model [33]. In contrast, ASF spread determined by network analysis in Estonia, Latvia, and Lithuania had a median ranging from 8.2 to 16.3 km/year, and a mean ranging from 13.9 to 33.5 km/year [51]. The disease had a lower estimated median spread in later reports in some European countries, such as Belgium, the Czech Republic, Estonia, Hungary, Latvia, Lithuania, and Poland [52]. Dissimilarities in the velocity of spread of ASF between regions are multifactorial and should be evaluated thoroughly and with caution. There are differences in the analysis methodology, as well as the type of transmission studied (natural and/or human-mediated). The natural spread of the disease in wild boar populations is influenced by the season, landscape structure, or demographic characteristics [51]. In the case of South Korea, anthropogenic factors might have played a crucial role in the spread of ASF, as well as the differences in wild boar abundance compared to Europe [13,14], aspects that will be explored in more detail later.

At the beginning of the epidemic, the disease spread to the south and east according to the directionality of the model (Fig 2), which is consistent with other analyses of its expansion [14,54]. Particularly, our results coincide with those of Ito *et al.* 2024, in which a directional distribution analysis with a three-month interval was performed [54]. The early directionality and restrained spread of ASF could be explained by the more urbanized and fragmented landscape, including the installation of a three-layer fencing system by the South Korean government to prevent the spread of ASF in wild boar, along with other control measures [9,14,55]. Although fencing did not stop the southward spread of the disease in South Korea, one study indicated that it helped to mitigate the spread of ASF in the region [7,55]. Fencing proved to be efficacious in Belgium. However, in Belgium, the introduction of the disease was highly localized, and early detection and control measures were implemented [50]. Moreover, the wild boar population density in the affected area may have been lower than in other ASF incursions. Long fences, such as the ones placed in South Korea [9,14], are usually inefficient in controlling wild boar movements, even more if they are not wild-boar-proof fences due to their fast degradation and costly maintenance [56]. The rapid expansion of the disease in the east (Fig 2) shows the importance of control measures. This area corresponds to the Taebaek mountain range, an elevated forested area, with high wild boar habitat suitability [7,15]. In this sense, a recent study associated the wild boar habitat suitability with ASF velocity in South Korea [49]. Several factors might be attributed to the dispersion of the disease in those areas. Firstly, the lack of natural and artificial fragmentation of the habitat of wild boar due to the lack of wide roads with considerable traffic or major rivers allows animals to move and spread freely [7,15]. To the authors’ knowledge there is no precise estimation of the density of this species in the area, although it could be high due to the high estimated density in human-inhabited areas [14], and the lack of large natural predators [57]. High animal density facilitates the onset of an infection in a given naïve population [58]. Moreover, the challenges in the implementation of control measures, such as carcass removal and population control can favor the expansion of the disease with scarce human intervention [7,9,50]. Carcass removal has been pointed out to be an effective control measure, since in its absence, ASFV-contaminated carcasses serve as a source for indirect transmission as well for the environmental contamination with ASFV [51,59,60]. Finally, whole-genome sequencing of ASF domestic outbreaks revealed distinct phylogenetic clusters with spatiotemporal associations [61]. Whether these genomic differences influence the biological characteristics of the virus, particularly its transmission, remains to be determined [61].

The model spatial resolution was sufficient to include wild boar home range size. This species has a relatively small home range, although several factors influence its extent, such as individual factors (sex, age, size), population density, habitat fragmentation, resources, as well as predator distribution [62]. The wild boar home range seldom exceeds 50 km^2^, although exploratory and dispersive movements could occasionally reach up to 100 km in half a year [58]. However, one limitation of the model was that the degree of carcass decomposition was not provided in the input data, which accounted for 90.46% of cases [25]. Hence, the date of the cases could introduce some uncertainty in the model outputs [50]. Yet, our TSA model presents a good fit for the grid-cell spatiotemporal distribution of ASF. In that sense, residuals complied with the assumption of normality and a mean of practically zero (Fig 3B). Positive residuals with extreme values were found in grid cells that reported relatively later than their adjacent ones (Figs 1 and 3A), presumably due to minor spread caused by recirculation of the disease in an already affected area that fell in an adjacent cell (Fig 3A). Another issue is related to the origin of ASF in the country. We considered the origin to be the first reported case in wild boar; however, due to the difficulty of surveillance on the border with North Korea the disease could have been circulating undetected, and several foci of introduction could have occurred [7,63].

On the other hand, negative residuals could be associated with human-mediated dispersion or silent spread of the disease due to under-surveillance. Some authors have pointed out the importance of human-mediated dispersion in South Korea that should be taken into consideration in the analysis of results [7,14]. In particular, culling methods, the delay in the placement of fences in some counties, as well as deficiency in biosafety measures during some control measures, such as wild boar population control, domestic pig culling, disinfection of vehicles, movement of people and carcass management, were highlighted to favored rather than hindered ASF dispersal [14]. In addition, the steep orography, limited habitat fragmentation and the presence of areas with very low or absent human activity contributed to uneven surveillance coverage in regions with highly suitable habitat for wild boar [7,15,54]. Due to the difficulties and heterogeneity in surveillance efforts in South Korea [63], ASFV could have been circulating before the official notification in those natural areas. However, since autumn 2022, Korean authorities improved the detection of wild boar carcasses by implementing new surveillance methods in orographic challenging areas such as, detection dogs and drones [54].

According to our model, the spread of ASF increased since August 2021 (Figs 2 and 4A), which is consistent with the original data (Fig 1). There was no clear seasonality in the velocity of spread, although autumn 2021 had more mean and median velocity. In line with this, behavioral seasonality was described in natural areas of Poland, which was more evident than in urban areas. In that study, autumn was the season with more activity duration and daily distance traveled in natural areas [62]. According to our results, the peak of the spread was in the summer of 2022 (Fig 4A); however, these high values should be analyzed with caution since they could be attributed to the edge effect of TSA models [20].

Focusing on the methodological aspects of this work, the calculation of the velocity differs from other previous works. Some studies used the inverse of the sum of the vectors obtained by substituting the coordinate values in the derivatives [22,23]. In a more recent approach, the speed was obtained as the inverse of the magnitude of the slope of disease spread, $\frac{\text{1}}{\sqrt{\left({\left(\frac{\partial T}{\partial X}\right)}^{\text{2}}+{\left(\frac{\partial T}{\partial Y}\right)}^{\text{2}}\right)}}$ [64]. We note that, considering benchmark problems (for instance, see the particular example proposed in S1 Appendix in S1 File), both approaches give poor approximations of the velocities whereas the formula proposed here gives reasonable estimations. However, due to the characteristics of the model, a limitation may arise when setting the upper threshold. The threshold velocity applied in this study (70 km/month) does not represent the natural spread of ASF in wild boar alone, as it was intentionally set high enough to also account for plausible human-mediated ASF spread [14] or uneven surveillance [54]. The sensitivity analysis showed that the median would be a better estimate, as it was more robust to variations in the upper threshold (Table 2).

## Conclusions

The estimated velocity of the disease in South Korea was considerably high with an overall median of 19.53 km/month, although it varied across the years included in the study. The geographical spread of ASF was more pronounced in natural areas which could be attributed to factors such as presumably higher wild boar density, reduced habitat fragmentation and challenges in implementing control measures [7]. Even though anthropogenic factors may have contributed to the dispersion of the disease in the country [14], similar or even greater biosecurity issues could be expected in other Asian countries where surveillance and wildlife control measures are often lacking [11,12]. Further studies, integrating additional modeling approaches, could be beneficial to identify the weight of natural and anthropogenic factors on the ASF velocity in South Korea. This study contributes to a better understanding of the potential spread of ASF in wild boar populations across Asia, thereby supporting the design of more effective control strategies. These control measures could also include the definition of wild boar vaccination zones as part of future vaccination campaigns, once a commercial vaccine becomes available, tailored to different ASF epidemiological contexts.

## Supporting information

S1 Table Best trend-surface analysis model (Eq. 1): coefficient, standard error and *p*-value. (DOCX)

S1 File Estimating velocities. (DOCX)
