# Supplementary material for: Estimating the velocity and direction of African Swine Fever spread in wild boar populations in South Korea using Trend-Surface Analysis
Source: PLoS One. 2026 Apr 2;21(4):e0346098. doi: 10.1371/journal.pone.0346098 (PMC13046126; doi:10.1371/journal.pone.0346098)
Supplement: S1 Table — (DOCX) [file pone.0346098.s001.docx]

Estimating the velocity and direction of African Swine Fever spread in wild boar populations in South Korea using Trend-Surface Analysis

**S1 Table. Best trend-surface analysis model (Eq. 1): coefficient, standard error and *p*-value.**

|  | **Coefficients no.** | **Coefficients** | **Standard Error** | ***p*-value** |
| --- | --- | --- | --- | --- |
| **Intercept** | *β*_0_ | 3.121346 | 6.38 x 10^-1^ | <0.001 |
| **y^2^** | *β*_1_ | 0.007135549 | 7.81 x 10^-4^ | <0.001 |
| **xy** | *β*_2_ | 0.005099335 | 5.61 x 10^-4^ | <0.001 |
| **x^3^** | *β*_3_ | 0.0000149365 | 2.54 x 10^-6^ | <0.001 |
| **y^3^** | *β*_4_ | 0.0000745064 | 8.77 x 10^-6^ | <0.001 |
| **x^2^y** | *β*_5_ | 0.0000790669 | 6.93 x 10^-6^ | <0.001 |
| **x^4^** | *β*_6_ | 0.0000000381 | 1.83 x 10^-8^ | <0.05 |
| **y^4^** | *β*_7_ | 0.0000001886 | 2.64 x 10^-8^ | <0.001 |
| **x^3^y** | *β*_8_ | 0.0000001329 | 3.84 x 10^-8^ | <0.001 |
| **x^2^y^2^** | *β*_9_ | 0.0000001461 | 2.33 x 10^-8^ | <0.001 |
