# Supplementary material for: Estimating the velocity and direction of African Swine Fever spread in wild boar populations in South Korea using Trend-Surface Analysis
Source: PLoS One. 2026 Apr 2;21(4):e0346098. doi: 10.1371/journal.pone.0346098 (PMC13046126; doi:10.1371/journal.pone.0346098)
Supplement: S1 File — (DOCX) [file pone.0346098.s002.docx]

Estimating the velocity and direction of African Swine Fever spread in wild boar populations in South Korea using Trend-Surface Analysis

## **S1 Appendix**

**Estimating velocities**

We consider a benchmark scenario where, at time 0 (s), ASF begins at the coordinate (0,0) m. By time 5 seconds, the disease has spread to the coordinate (2,3) m. In this case, we calculate the following values: $\partial T=5s$, $\partial X=2m$, and $\partial Y=3m$. Based on these values, the exact velocity, assuming straight-line propagation, is 0.72 m/s (3.6 meters covered in 5 seconds).

Applying the formula proposed in (Morrison & Zinszer, 2017), $1/{\sqrt{\left( \left( {\partial T}/{\partial X} \right)^{2}+\left( {\partial T}/{\partial Y} \right)^{2} \right)}}$, we estimate a velocity of 0.33 m/s. Similarly, using the formula proposed in (Moore, 1999), $1/{({\partial T}/{\partial X}+{\partial T}/{\partial Y})}$, we obtain a velocity of 0.24 m/s. Both values clearly underestimate the real velocity.

In contrast, by using the formula proposed in this work,
$\sqrt{\left( \frac{\partial X}{\partial T}\left( x,y \right) \right)^{2}+\left( \frac{\partial Y}{\partial T}\left( x,y \right) \right)^{2}},$ we calculate a velocity of 0.72 m/s, which closely matches the exact velocity.

This example demonstrates the accuracy of our approach and highlights why it should be considered.

**References**

1. Moore, D. A. (1999). Spatial diffusion of raccoon rabies in Pennsylvania, USA. Prev Vet Med, 40(1), 19-32. doi:10.1016/s0167-5877(99)00005-7
2. Morrison, K., & Zinszer, K. (2017). R package: outbreakvelocity. Retrieved from https://github.com/kathryntmorrison/outbreakvelocity
